# A Cadaveric Study to Define Morphology and Morphometry of Human Knee Menisci in the Region of Central India

**DOI:** 10.7759/cureus.41174

**Published:** 2023-06-30

**Authors:** Prashant Chaware, Brijesh Kumar, Sumit Patil, Venkata Surya, Bertha Rathinam, Kusum Gandhi, Manmohan Patel

**Affiliations:** 1 Anatomy, All India Institute of Medical Sciences, Bhopal, Bhopal, IND; 2 Anatomy, Kasturba Medical College, Manipal, Manipal, IND

**Keywords:** knee arthroscopy, meniscal injuries, meniscal morphometry, meniscal morphology, knee joint

## Abstract

Introduction

The medial and lateral menisci of the knee joint are the functional unit that helps to increase the depth of articular surfaces on the head of a tibia for the reception of femoral condyles. Menisci are important for the distribution of load and thus help to reduce stress on the knee joint. The anatomical knowledge of morphology and morphometry of menisci is vital while performing surgeries in cases of meniscal injury. The study aimed to define the variational anatomy of the menisci of the knee joint.

Material and Methods

Ninety-six cadaveric knees of 48 cadavers were included in the study. Different shapes of both; lateral and medial menisci were recorded. The peripheral lengths and inner lengths of the menisci were measured with the nonelastic cotton thread. Menisci were divided into three parts; anterior, middle, and posterior. The maximum width and thickness of each part of the menisci were measured and documented. Distance between the medial and lateral meniscus in each knee was measured at the anterior and posterior ends. The most anterior and the most posterior part of each meniscus was determined and the distance between these parts of each meniscus was measured and recorded as the distance between two horns.

Results

Four morphological types of menisci were found; in medial menisci, the most common was crescent‑shaped (53%), and in lateral menisci, the most common shape was c‑shape (62.5%). The average peripheral length of the medial menisci was 92.0 mm and the lateral menisci was 96.08 mm while the average inner length of the medial and lateral meniscus was 56.19 mm and 58.92 mm respectively. The anterior third of the medial as well as lateral meniscus was thinnest while the posterior third was thickest. The width of the medial menisci was less at the anterior end and was more at the posterior ends while the width of the lateral meniscus was almost the same at the anterior, middle, and posterior ends.

Conclusion

The findings of the present study will be helpful for surgeons while planning and performing surgical procedures and for anatomists during routine teaching.

## Introduction

The menisci of the knee joint are a pair of wedge-shaped semilunar cartilages which are present between the femoral condyles and tibial plateaus [1]. They help to increase the depth of the articular fossae on the head of the tibia for the reception of the femoral condyles [2-4]. They provide mechanical protection to the articular surface of the tibia as they reduce the stress on the knee joint and help in preventing osteoarthritis [5-6].

The menisci perform important mechanical functions and are prone to injury. They may get damaged during rotation or bending of the knee joint, due to degenerative changes, or as a spontaneous injury due to progressive structural failure. This spontaneous injury is unrelated to any kind of wear and tear or trauma [7].

With the development of newer imaging modalities like magnetic resonance imaging (MRI), visualization of intraarticular structures under a microscope (arthroscopy) and computed tomography (CT) scan, anatomic abnormalities and variations of the intraarticular structures of knee joints such as menisci have become significant. Their variant patterns are important for clinical diagnosis and surgical repairs [8]. Repair of meniscus is the preferred treatment for ruptured meniscus. In cases of irreversible damage of the meniscus, partial meniscectomy is preferred over total to minimize its loss [6]. For the success of these treatment modalities, precise anatomy of the menisci of the knee joints is necessary. Kohn et. al. [9] have mentioned in their study that for the replacement of menisci of knee joints, accurate data on its shape and size is necessary. Also, the variations of form and in particular of thickness and width of menisci can determine the possibility and the kind of injury [10]. It is important for surgeons and radiologists who are involved in the treatment and diagnosis of meniscal injuries to know the variational anatomy of menisci [7].

The current study was conducted to estimate the incidence of various shapes and to report the variations in morphometry of medial and lateral menisci in the central Indian population.

## Materials and methods

The study was conducted in the Department of Anatomy, All India Institute of Medical Sciences (AIIMS), Bhopal after due ethical clearance (LOP/2015/IM 0062). Ninety-six cadaveric knees of 48 cadavers aged between 55 to 88 years were included in the study. Out of 48, only 11 were female cadavers. All cadaveric knees were fixed in 10% formaldehyde.

The longitudinal skin incision was taken, subcutaneous soft tissues were cleared, patellar ligament and the collateral ligaments were cut transversely. Patella was pulled upwards to expose the joint cavity. To visualize the menisci, a longitudinal incision was taken on each side of the joint capsule, and it was retracted downwards. To approach the menisci, intra-articular ligaments were cut, and surrounding connective tissue and adipose tissue was cleaned. Different shapes of both; lateral and medial menisci were observed and recorded. To record the morphometric data, a digital vernier caliper was used. To measure peripheral (outer) lengths as well as inner lengths of menisci, cotton threads were used. The distance between most anterior parts of the meniscus to the most posterior part of it, along the outer margin was considered as ‘peripheral length’ while the distance between most anterior parts of the meniscus to the most posterior part of it, along the inner margin was considered as as ‘inner length’. To measure the peripheral length of the meniscus, metallic pins were inserted along the outer margin of the meniscus so that the thread can be placed by taking support of the pins. Then the length of the thread was measured by the vernier calipers. The same method was followed to measure the inner length of the meniscus. Taking reference to peripheral and inner lengths, menisci were divided into three parts; anterior, middle, and posterior. The maximum width and maximum thickness of each part of the menisci were measured at three sites, anterior, middle, and posterior part and documented. All the measurements were recorded using Zhart digital venire calipers (range 0.01 mm to 150 mm). All findings were tabulated and statistical analysis was done by MS Excel.

## Results

In this study, four shapes of menisci were observed, crescentic-shaped, U-shaped, V-shaped, and C shaped.

**Figure 1:**
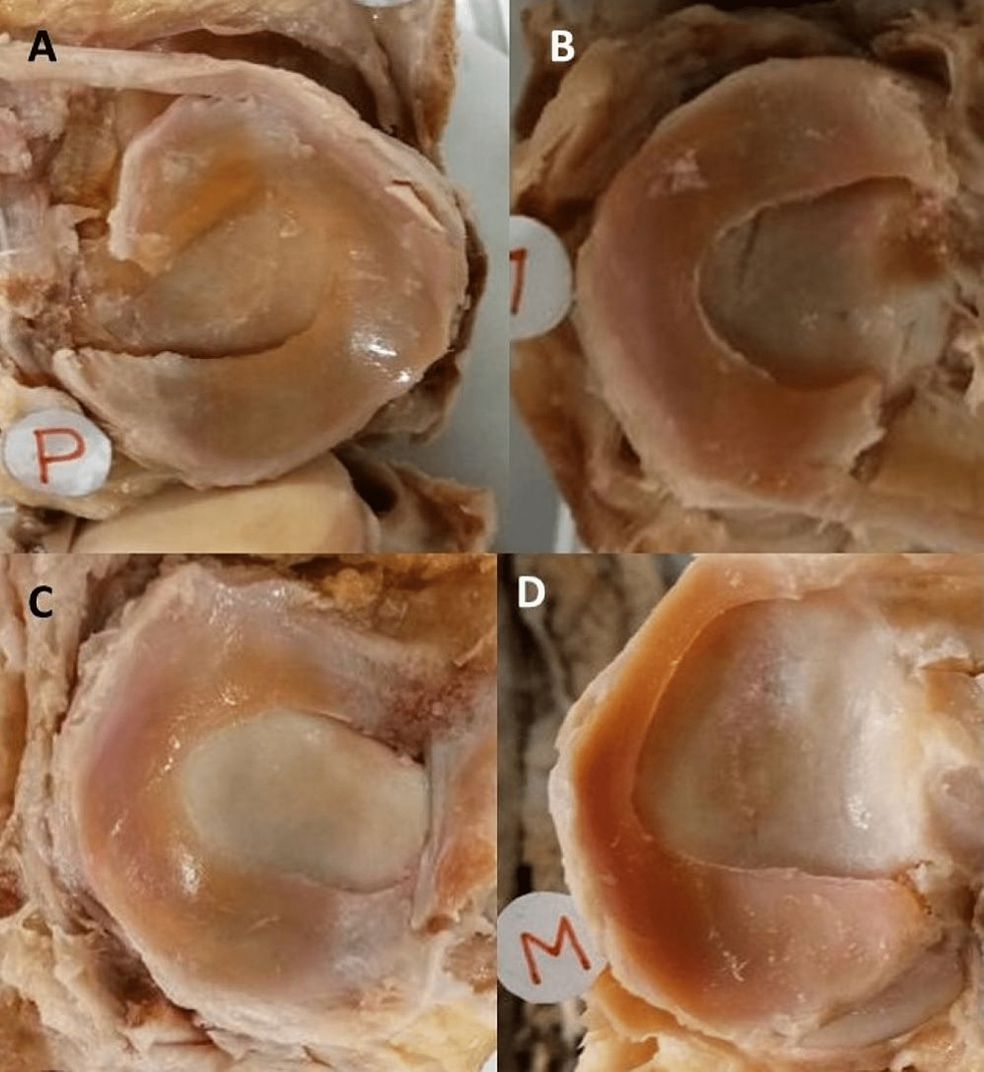
Different shapes of menisci. A - crescentic shaped; B -  'C' shaped; C - 'U' shaped; D - 'V' Shaped P: Posterior, M: Medial

The details are as follows (Figure 1): left-sided medial meniscus - crescentic shape (52%), U shape (38%), V shape (10%), right-sided medial meniscus - crescentic shape (54%), U shape (39%), V shape (7%), left-sided lateral meniscus - C shape (63%), crescentic shape (37%), right-sided lateral meniscus - C shape (62 %), crescentic shape (38 %).

The average peripheral and inner lengths of medial and lateral menisci on the left and right side, maximum width and maximum thickness of menisci, and distance between anterior and posterior horns of medial as well as lateral menisci were as per Table 1.

**Table 1:** Various parameters of medial and lateral menisci measured in millimeter (mm) PL: peripheral length, IL: inner Length, T1: thickness at the anterior end, T2: thickness at the middle end, T3: thickness at the posterior end, W1: width at anterior end; W2-Width at the middle end, W3: width at the posterior end, Dist. Btw horns: distance between anterior and posterior horns, mm: millimeter, RMM: right medial meniscus, LMM: left medial meniscus, RLM: right lateral meniscus, LLM: left lateral meniscus

	PL (mm)	IL (mm)	Thickness (mm)			Width (mm)			Dist. Btw horns (mm)
			T1	T2	T3	W1	W2	W3	
RMM	92.32 ±5.48	56.52 ±3.74	4.65 ±0.64	5.28 ± 0.79	5.78 ±1.22	7.28 ± 0.97	8.48 ± 1.27	13.44 ±2.89	22.28 ±3.85
LMM	91.68 ±5.94	55.87 ±3.11	4.24 ±0.94	5.72 ± 0.92	6.12 ±1.59	6.83 ± 1.03	7.76 ± 1.47	14.13 ±2.35	21.94 ±3.57
RLM	96.44 ±6.86	58.52 ±3.86	5.18 ±0.85	6.15± 0.98	6.94 ±1.74	10.40 ±2.45	11.44 ±3.65	10.73 ±3.74	13.47 ±2.86
LLM	95.73 ±7.21	59.33 ±3.77	5.64 ±1.36	6.86 ±1.17	7.24 ±1.45	10.96 ±2.34	12.04 ±3.45	11.06 ±2.96	13.26 ±2.62

The peripheral length of the lateral meniscus (96.085±7.03) was more than that of the medial meniscus (92±5.71) but the difference was not significant (p-value≥ 0.05) statistically. The distance between the anterior and posterior horn of the medial meniscus (22.11 ± 3.71 mm) was significantly higher than that of the lateral meniscus (13.36 ± 2.74 mm) having a p-value of 0.0001. From Table 1, it can be observed that the thickness of both menisci was more at the posterior end as compared with the anterior and middle ends and the difference was statistically significant in the parameters of the anterior and posterior ends. The width of the medial meniscus was more at the posterior end as compared with the anterior and middle ends while the width of the lateral meniscus was almost the same at the anterior, middle, and posterior ends.

## Discussion

The knowledge of the variational anatomy of menisci is significant as injury to them leads to significant morbidity related to locomotory function. Tears of menisci are common, most of which occur in the avascular inner zones and seldom heal spontaneously. Peripheral tears have the potential to heal satisfactorily if repaired surgically [11]. So, studies to document the morphological and morphometric features of knee menisci are very much needed.

In the present study, variations in the morphological features of lateral and medial menisci were observed and recorded. Medial menisci were crescentic-shaped (53%), U-shaped (38.5 %), and V-shaped (11.50 %), while lateral menisci were C shaped (62.5 %), and crescentic (37.5%) shaped. The findings of our studies are in accordance with the findings of B.V. Murlimanju et al. [12] who reported shapes of lateral menisci as C shaped (61.1%) and crescentic (38.9%) while shapes of medial menisci as crescentic shaped (50%), V-shaped (38.9%) and U shaped (11.1%). Parsons et al. [13] reported in their study that the medial menisci are always crescent shaped whereas the lateral menisci may be either crescent or disc-shaped. The medial menisci were described by Flick et al. [14] as half, two-thirds, or three‑fourth ellipse, while the lateral menisci were compared to an almost complete circle. However, Charles et al. [15] classified the menisci based on the relative size of the anterior and posterior cornua and the degree of curvature.

In the present study, the average peripheral length of the medial menisci was 92.0 mm and the lateral menisci was 96.08 mm while the average inner length of the medial and lateral meniscus was 56.19 mm and 58.92 mm respectively. Our findings are in accordance with the study of Braz et al. [7] who reported peripheral length of the medial menisci was 91.85 mm and lateral menisci 92.80 mm and the inner length of the medial menisci 55.16 mm and the lateral menisci 57.84. As mentioned in studies conducted by researchers the medial meniscus was larger than the lateral, but these authors haven’t mentioned the values of their measurements [7]. It was found in the present study that the distance of the anterior horn of the medial meniscus from its posterior horn was 22.11 ± 3.71 mm while the distance of the anterior horn of the lateral meniscus from its posterior horn was 13.36 ± 2.74 mm. Our findings are in accordance with the study of Braz et al. [7] who reported distance between the anterior horn of the medial meniscus from its posterior horn was 25.88 mm while the distance between the anterior horn of the lateral meniscus from its posterior horn was 12.55 mm and also with findings of Kohn et al. [9] who described that the distance between horns of the lateral meniscus is smaller compared to that of the medial meniscus.

From Table 2, it can be seen that the thickness and width measurements of menisci in the present study are similar to the findings of the previous studies. It was observed in the present study that, the anterior third of the medial as well as lateral meniscus was thinnest while the posterior third was thickest which connects with the study done by Almeida et al. [10]. Braz et al. [7], Bhatt et al. [16] and Hathila et al. [17]. The present study reports that the width of the medial menisci was less at the anterior end and was more at the posterior ends while the width of the lateral meniscus was almost the same at the anterior, middle, and posterior ends. These findings were similar to what was reported in the previous studies [7,10,16-17].

**Table 2:** Comparison of measurements of parameters in the present study with previous studies mm: millimeters

Parameters	Present study	Almeida et al. [10]	Braz et al. [7]	Bhatt et al. [16]	Hathila et al. [17]
Thickness of medial menisci (mm)					
Anterior	4.45±0.78	5.92±1.37	6.17±1.68	5.82±1.44	6.21±0.60
Middle	5.50±0.83	5.31±1.06	6.31±1.73	5.64±1.26	6.18±0.55
Posterior	5.95±1.39	5.91±1.13	5.18±1.55	5.86±1.06	6.30±0.42
Width of medial menisci (mm)					
Anterior	7.05±0.95	9.02±1.59	7.68±1.36	8.78±2.12	9.05±0.70
Middle	8.12±1.37	12.16±2.58	9.32±2.24	12.08±2.52	11.10±0.45
Posterior	13.78±2.56	17.37±2.22	14.96±2.66	16.46±2.18	15.39±0.80
Thickness of lateral menisci (mm)					
Anterior	5.41±1.14	3.71±1.15	4.40±0.83	3.70±1.52	4.15±0.50
Middle	6.55±1.06	6.10±1.04	6.52±1.81	5.78±1.22	5.90±0.61
Posterior	7.09±1.59	5.29±0.78	5.46±1.19	5.20±0.98	5.63±0.60
Width of lateral menisci (mm)					
Anterior	8.00±2.38	11.86±1.81	11.32±1.46	11.30±1.30	11.82±0.81
Middle	11.54±3.56	11.97±2.56	11.16±1.64	11.66±1.48	12.53±0.72
Posterior	12.65±3.46	11.44±1.07	11.67±1.54	11.50±1.34	12.03±0.80

Meniscal injury due to any reason leads to knee pain and osteoarthritis affecting the day-to-day life functioning of an individual. So, the knowledge of morphology and morphometry of medial as well as lateral menisci is very essential for the orthopedic surgeon and the findings of the present study will add to the knowledge of treating surgeons about variant features of menisci of the knee joint.

The study had limitations in that it was conducted on 96 knees obtained from 48 cadavers of which only 11 were female cadavers. So, we were not able to comment on differences in males and females regarding the anatomy of menisci. All knees were preserved in 10% formaldehyde over a period of four to five years. The results would have been more accurate if a study had been done on fresh frozen cadavers.

## Conclusions

Medial and lateral menisci are of different shapes and show variant anatomy at the anterior and posterior ends. Medial and lateral menisci have different inner lengths and peripheral lengths. It is very important to keep in mind the variant anatomy of menisci while planning various procedures on knee joints including arthroscopy. The findings of the present study will be helpful for surgeons while planning and performing surgical procedures and for anatomists during routine teaching.
